# Value of point-of-care ultrasonography compared with computed tomography scan in detecting potential life-threatening conditions in blunt chest trauma patients

**DOI:** 10.1186/s13089-020-00183-6

**Published:** 2020-08-04

**Authors:** Amirhosein Jahanshir, Shida Mohajer Moghari, Ayat Ahmadi, Pejman Z. Moghadam, Maryam Bahreini

**Affiliations:** 1 Emergency Department, Shariati Hospital, Tehran University of Medical Sciences, Tehran, Iran; 2 Tehran University of Medical Sciences, Tehran, Iran; 3 Knowledge Utilization Research Center, Tehran University of Medical Sciences, Tehran, Iran; 4 Trust ST1/2 in Emergency Medicine, Mid Essex NHS Trust Broomfield Hospital, Chelmsford, Essex, UK; 5 Department of Emergency Medicine, Sina Trauma and Surgery Research Center, Sina Hospital, Tehran University of Medical Sciences, Tehran, Iran

**Keywords:** Chest injury, Computed tomography, Emergency, Hemothorax, Lung injury, Pneumothorax, Predictive value of tests, Ultrasonography

## Abstract

**Background:**

Ultrasonography is a suitable modality that can potentially improve patient care, saving time and lives.

**Purpose:**

This article has evaluated the caveats and pitfalls of point-of-care ultrasonography in the diagnosis of pneumothorax, hemothorax and contusion.

**Materials and methods:**

This prospective study was performed in 157 patients with blunt chest trauma in 3 university hospitals. Ultrasonography was performed by 2 board-certified emergency medicine specialists and an emergency medicine resident PGY-3 after passing the training process successfully.

**Results:**

The false-negative cases were not significantly correlated with accompanying traumatic injuries. Lung ultrasonography accompanied by chest physical examination show accuracy 91.8. Point-of-care ultrasonography (PoCUS) showed sensitivity 75.0%, specificity 100%, positive-predictive value (PPV) of 100% and a negative-predictive value (NPV) of 94.9% for the diagnosis of pneumothorax. For hemothorax, bedside PoCUS had a sensitivity of 45.4%, specificity of 100%, PPV of 100% and NPV of 91.8%. PoCUS was assessed 58.1% sensitive and 100% specific for detecting lung contusion with positive-predictive value (PPV) of 100% and a negative-predictive value (NPV) of 86.3%. Performing US resulted in no false-positive cases.

**Conclusions:**

Point-of-care ultrasonography was highly sensitive to detect pneumothorax and can be beneficial for the disposition of stable patients and to detect PTX in unstable patients before transferring to the operating room. It is also moderately appropriate for the diagnosis of hemothorax and lung contusion compared to the gold standard, CT scan. It is essential to consider the false-negative and false-positive instances of lung ultrasound in various situations to enhance management and disposition of blunt thoracic injuries.

## Introduction

### Background

Thoracic injuries account for 20 to 25% of the trauma mortality which can be preventable by timely diagnosis. Despite of undesirable low sensitivity, chest X-ray (CXR) has remained one of the adjuncts for evaluation of chest trauma. Bedside ultrasonography (US) has an important role in primary and advanced trauma life support (ATLS) due to higher sensitivity and specificity to detect of pneumothorax (PTX) and hemothorax (HTX) [1–3]. However, lung contusion, a common injury in 30–75% of blunt chest trauma, may remain undiagnosed by radiography or ultrasonography depending on the extent and intensity [4]. Besides, computed tomography (CT) scan is the standard modality for the assessment of lung parenchymal injuries. Yet it is time-consuming, expensive and there are concerns about radiation exposure and patient hemodynamic instability during transfer [1]. In this context, multiple trauma patients often undergo CT scan in trauma centers; however, the presence of clinical injuries that require emergent operative management in unstable patients such as brain lateralizing signs, necessitates the use of extended focused assessment with sonography in trauma (eFAST) bedside exam and bypassing torso CT scan. This may also remain true for the stable and selective population of patients with high risk mechanism of injury to reduce the risk of radiation. Based on the possibility of diagnosing potential life-threatening conditions in traumatic injuries by US, revising the Advanced Trauma Life Support (ATLS) guidelines have been proposed to incorporate eFAST in the work-up of high-energy trauma patients [5].

### Goals of investigation

Ultrasound may be overused without sufficient applicability in some situations. As the published papers were unable to find a unified answer for this issue, we discuss pitfalls and caveats to point-of-care lung ultrasound in various traumatic chest injuries, analyze false-negative and false-positive cases and highlight the growing topic of the point-of-care ultrasound (PoCUS) despite its common diagnostic limitations. Moreover, the diagnostic accuracy of ultrasonography was estimated in the detection of pneumothorax, hemothorax and contusion simultaneously in comparison with the gold standard, CT-scan.

## Materials and methods

### Selection of participants

Patients with blunt chest trauma were included if they were 18 years or older with acute chest trauma defined as the isolated blunt trauma to the chest or back and also multiple trauma patients involving chest trauma. All chest trauma patients were recruited in the study including unconscious or apneic patients, or individuals with open chest injuries. All participants consented to enter the study.

Patient with old chest trauma, past history of lung fibrosis or if they were not planned or assented to CT scan (except for clinically unstable patients who underwent chest tube insertion) were excluded.

### Study design and setting

This longitudinal study was performed on 157 patients with blunt chest trauma in 3 university hospitals including a level I and 2 level II trauma centers with approximately 2200–3850 trauma patients per year. Bedside chest ultrasonography was performed by 2 board-certified emergency medicine specialists, certified from the Board Committee of Emergency Medicine of the country, and an emergency medicine resident PGY-3 (SM), in the third year of the residency program. The PGY-3 emergency medicine resident required to perform 50 successful ultrasound scans in this field prior to this study. These operators were blinded to the findings brought up by chest exam and radiography and were not involved in patient management. Patients were included by convenience sampling in the operators’ clinical shifts 24/7 in the emergency department.

### Interventions

As a common practice, the patients underwent the standard of care according to the ATLS guideline. Standard chest physical exam was performed routinely and documented consisting of tachypnea, abrasion or laceration, emphysema, rib tenderness or crepitation, hypotension, oxygen desaturation, stridor and other factors discussed in Table 1. Then, chest US was performed prior to imaging. Trauma patients commonly underwent supine CXR although in selected stable patients with definite intact spine, upright films might have been ordered up to the clinician’s judgement and patient’s condition. Chest X-ray was planned except for patients who were suspected for tension pneumothorax or massive hemothorax by physical exam and bedside ultrasonography. If rush of air or 100 ml of blood was detected during tube thoracostomy, the diagnosis became confirmed. These patients might undergo CT scan for further trauma evaluation if they became clinically and vitally stable; otherwise, because of the definite diagnosis, they were included in the study without CT scan. CTs were homogeneous by the technique applied and performed using 16-slice multidetector CT scanner (Philips Brilliance, Emotion, and Ingenuity) with slice width of 3 mm. Board-certified emergency physicians, who were blinded to the study, interpreted them.

**Table 1:** The diagnostic findings applied to assess traumatic chest injuries in physical exam and ultrasonography

Assessment	Definition	Simple pneumothorax	Tension pneumothorax	Hemothorax	Massive hemothorax	Pulmonary contusion
Findings of physical examination						
Inspection	Chest expansion	Nl-↓	↓	↓	↓	Nl
	Trachea	Nl	Deviated	Nl	Deviated	Nl
	Jugular vein pressure	Nl	↑	Nl	↓	Nl
Percussion	The sound of striking 2 fingers on intercostal spaces	Nl-hyperresonance	Hyperresonance	Nl-dull	Dull	Nl
Auscultation	To hear both sides comparatively and note sounds’ quality	↓	Nl-dull	Nl-↓	↓	Nl-crackles
Findings of ultrasonography						
Pleural sliding	The shimmering movement of parietal pleura during inspiration	Lost in injured zone	Lost	Nl	May be Nl	Maybe falsely ↓
Seashore sign	Normal lung M-mode of sandy appearance above and parallel lines below	Lost in injured zone	Lost	Nl	May be Nl	May be Nl
Barcode/stratosphere sign	Abnormal M-mode showing multiple parallel lines	+ in injured zone	+	–	–	May be falsely +
Lung point	The interface of normal lung and pneumothorax area in B-M mode	May be +	Often -	–	–	–
Sinusoid sign	The sinusoidal movement of the collapsed lung in the pleural fluid	–	–	+	+	–
V-line	Echogenic vertebral line with posterior shadow due to the transmission of ultrasound waves through the pleural fluid	–	–	May be +	+	–
B-lines/comet tails	Vertical echogenic artifact lines from the pleura to the screen edge, if multiple, resulting from alveolo-interstitial syndrome (rocket sign)	Lost	Lost	Nl	May be Nl	↑
Peripheral parenchymal lesions	Lung hepatization with subpleural hypoechoic foci and pleural line gap	–	–	–	–	+

Based on the discretion of the treating physician including clinical judgement in conjunction with the NEXUS chest trauma criteria, stable patients underwent chest computed tomography within 6 h of their trauma. As standard management in our setting, the time elapsed from the chest physical exam and CXR to chest CT was not more than 90 min. The chest trauma injuries were confirmed either by CT scan or by performing an emergent thoracostomy [6]. In our trauma centers, CT scan is frequently used and accessible 24/7. Generally, nearly asymptomatic patients with isolated occult PTX, small amount unilateral pneumothorax, hemothorax or contusion were observed to discharge without intervention according to our ED management protocols.

### PoCUS protocol

PoCUS was performed by Medison 8 (SONOACE), SonoSite Edge II and Samsung UGEO HM70A and 3–8 MHZ and 7–15 MHZ transducers. Each lung was scanned at least in 4 spaces for the evidence of pneumothorax, hemothorax and lung contusion by B and M modes: the probe was placed in the 2nd to 4th intercostal spaces in midclavicular and parasternal lines and the 6th or 7th intercostal space in the mid-axillary line in the supine position.

Subsequently, the low-frequency probe was positioned in the 5th to 6th right anterior to mid-axillary region and 7th to 8th left mid to posterior axillary region to find hemothorax as part of the eFAST exam [7]. Each bedside scan was performed routinely in 3–5 min.

The detailed diagnostic findings of chest traumatic injuries including simple and tension PTX, simple and massive HTX and pulmonary contusion determined by ultrasonography and physical exam are presented in Table 1. Table 2 demonstrates how we conducted our study using CXR, ultrasonography (US) and CT scan for traumatic lung injuries.

**Table 2:** The diagnostic criteria of chest X-ray, US and CT scan to determine lung injuries in our settings

Diagnostic *criteria*	PTX	HTX	Contusion
Chest X-ray	A thin dense line as the visceral pleural edge separating from the parietal pleura, the distance between the 2 pleurae is radiolucent usually accumulating in the periphery and sometimes deep lucent lateral costophrenic angle	In the presence of sufficient amount of blood, the involved hemithorax is hazy. If upright film is performed, the costophrenic sulcus become blunted.	Alveolar infiltration, irregular patchy consolidation that usually evolve within the first 6 h
US	Absence of lung sliding and the presence of stratosphere sign and the absence of B lines with or without “lung point”	Dependent collection above diaphragm with or without the sinusoidal movement of collapsed lung in inspiration in the B and M mode scan, the presence of V line	Three B lines in at least 2 zone scans, alveolo-interstitial syndrome (AIS), peripheral parenchymal lesions (PPL) and punctiform hyperechoic lesions without respiratory alterations
CT scan	In the pulmonary window, the existence of air in pleural space that may collapse the adjacent lung	Blood in the pleural space with Hounsfield Unit of about 45–65 in the mediastinal window	An interstitial or alveolar lung injury, usually focal and non-segmental peripheral opacification in lung parenchyma in lower lobes

*PTX* pneumothorax, *HTX* hemothorax, *US* ultrasonography, *CT* computed tomography

### Outcomes

This study aimed to widely assess the thoracic injuries by point-of-care ultrasonography in the emergency department compared to the computed tomography findings and interpreted false-positive and negative results.

A questionnaire was designed containing demographic data such as age, gender, trauma mechanism and detailed information on physical exam, ultrasonography and CT scan to detect thoracic injuries. As different scales are available to score injury severity with various accuracies and no definitive superiority, we report injuries according to the injury severity scale (ISS) [8, 9].

### Analysis

Data were analyzed by SPSS, version 22.0. Chicago, IL, USA. Sensitivity, specificity, positive-predictive value (PPV) and negative-predictive value (NPV) of different diagnostic modalities were assessed. Besides, univariate regression analysis was performed to assess single factors that are correlated with false-negative results including some related injuries or multisystem injuries. The type-I error 0.05 was considered significant and the confidence intervals (CI 95%) were reported. Sample size calculation met 80% power for 95% sensitivity. False-negative episodes were analyzed with regard to the results of physical examination, CT scan and outcome details to assess whether they were clinically significant or not. In this context, false-negative cases are categorized by findings such as occult PTX, subcutaneous emphysema, which was analyzed in these cases as a confounder to ultrasound results, and also the clinical decision for placing tube thoracostomy.

### Ethical considerations

The protocol of study has been reviewed and approved by the University of Medical Sciences Institutional Review Board and the ethical committee. All procedures were in accordance with the ethical standards of the institutional and national research committee and with the 1964 Helsinki declaration and its later ethical standards. Informed consent was obtained from the conscious participants and the relatives of critically ill patients.

## Results

### Characteristics of study subjects

**Fig. 1:**
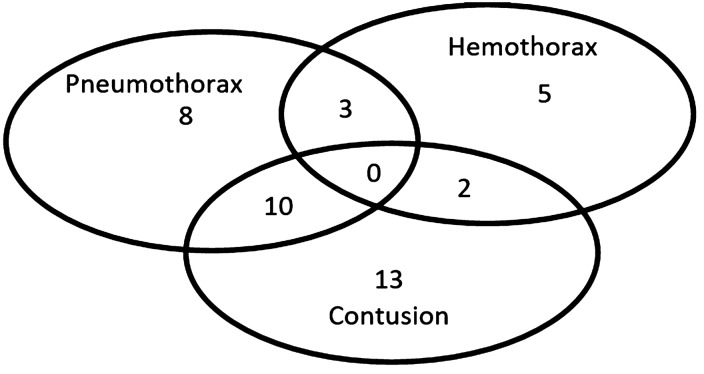
The Venn diagram of the positive samples for pneumothorax, hemothorax and lung contusion detected by ultrasonography

In this study, of 160 patients, 157 agents with blunt chest trauma were enrolled after obtaining informed consent, of which 134 (85.4%) were men. Three patients were excluded, 1 was underaged and 2 were not willing to undergo the study. The mean age was 38.3 (SD: 18.57) years. Motor vehicle collision (*n* = 42, 26.8%) and falling (*n* = 43, 27.4%) were the most common injury mechanisms. Among all patients, 94 (59.87%) cases had injury severity score 10–50 while 19 (12.10%) cases had ISS more than 50. Furthermore, 56 (35.6%) of patients had pneumothorax (*n* = 21, 13.3%), hemothorax (*n* = 10, 6.3%) or contusion (*n* = 25, 15.9%). The Venn diagram of samples of different diagnoses by PoCUS is presented in Fig. 1.

### Main results

The sensitivity, specificity, PPV, NPV and accuracy of point-of-care ultrasonography for the diagnosis of blunt pneumothorax, hemothorax and pulmonary contusion are shown in Table 3. The US results for pneumothorax was 75.0% (CI 55.1–89.0), while it was 45.4% (CI 24.4–67.8) and 58.1% (CI 42.1–73.3) for hemothorax and pulmonary contusion, respectively. Performing US resulted in no false-positive cases (specificity = 100%), but when it was combined with physical examination, 9 cases were falsely diagnosed as positive (negative false-positive rate = 9.2%). Rib fracture was present in 25 patients (15.9%). Three patients (1.9%) were intubated as a result of other traumatic injuries. None needed resuscitative or urgent thoracotomy.

**Table 3:** Sensitivity, specificity, PPV, NPV and the accuracy and point-of-care ultrasound in the diagnosis of blunt chest injuries

Type of injury	Diagnostic tool	Analysis	Point estimate	95% CI^a^
Pneumothorax	Sonography	Sensitivity, %	75.0	55.1–89.0
		Specificity, %	100	97.2–100
		PPV%	100	83.9–100
		NPV%	94.9	89.7–97.9
		Accuracy%	95.5	91.0–98.2
Hemothorax		Sensitivity, %	45.4	24.4–67.8
		Specificity, %	100	97.3–100
		PPV%	100	69.2–100
		NPV%	91.8	86.2–95.7
		Accuracy%	92.4	87.0–96.0
Contusion		Sensitivity, %	58.1	42.1–73.3
		Specificity, %	100	96.8–100
		PPV%	100	86.3–100
		NPV%	86.3	79.3–91.7
		Accuracy%	88.5	82.5–93.1
Pneumothorax, hemothorax and contusion	Physical exam and sonography	Sensitivity, %	91.5	81.3–97.2
		Specificity, %	90.8	83.3–95.7
		PPV	85.7	74.6–93.3
		NPV	94.7	88.0–98.3
		Accuracy	91.8	85.5–95.0

^a^Binomial exact calculation

### Description of false-negative cases

To elucidate why US missed PTX in 7 cases, false-negative results were reviewed. Of 157 enrolled participants, 28 (17.83%) patients had pneumothorax in CT scan. Seven patients were not diagnosed by US of whom, 3 patients had concomitant subcutaneous emphysema and another 2 had occult PTX (*P* = 0.000). Regarding false-negative patient outcomes, 2 (of 7 false-negative results) were discharged with follow-up, 5 patients including those with subcutaneous emphysema required tube thoracotomy and 4 out of those 5 patients were suspected to have pneumothorax on physical exam. Ultrasound was not helpful to diagnose hemothorax in 12 out of 21 CT confirmed patients. Besides, CT scan confirmed lung contusion in 43 patients but 18 cases were not diagnosed by bedside ultrasonography of whom, 8 individuals had definite concomitant pneumothorax. There were too few false-negative cases to quantitatively appraise its correlated determinant factors in univariable and multivariable (logistic regression) analysis. However, occult pneumothorax and also the presence of concomitant hemothorax or lung contusion were more frequently seen among false-negative results than the state of clinical instability or the presence of multisystem injuries. Overall, 2 patients were not cooperative to undergo lung US and CT scan and were excluded. Only 3 patients acquired Glasgow Coma Scale under 9.

Furthermore, the diagnostic performance of ultrasound alone and positive physical exam in conjunction with ultrasound was assessed in comparison with CT scan and exhibited in Table 3.

There was no false-positive case in our study for traumatic lung injuries. The possible causes for false results of ultrasonography are shown in Table 4.

**Table 4:** Possible suggested causes for false-positive and negative results of bedside ultrasonography for the diagnosis of traumatic chest injuries

Traumatic chest injury	Missed diagnoses	Causes
Pneumothorax	False positive	Achalasia
		Pleural adhesions
		Phrenic nerve palsy
		Pulmonary fibrosis
		Pulmonary atelectasis
		Cardiopulmonary arrest
		Congestive heart failure
		Right main bronchus intubation
		Subcutaneous chest emphysema
		Acute respiratory distress syndrome
		Significant concomitant lung contusion
		Bullous emphysema/pulmonary blebs
		Chronic obstructive pulmonary disease
		High positive end expiratory pressure-ventilated patients
	False negative	Lung remained in contact with the chest wall despite PTX
		Operator, machine or transducer conditions
		Cardiac movement with left lung sliding
		Subcutaneous chest emphysema
		Occult small pneumothorax
Hemothorax	False positive	Pleural effusion differential diagnoses
	False negative	Minimal amount hemothorax
		Subcutaneous emphysema
Lung contusion	False positive	Pulmonary fibrosis
		Cardiogenic pulmonary edema
		Acute respiratory distress syndrome
	False negative	Subcutaneous emphysema
		Inaccessible to ultrasound (retrosternal, paravertebral, etc.)

*PTX* pneumothorax

## Discussion

The concrete indications for chest imaging have not yet been well established. There are some exclusion criteria for reducing CXR requests [10]. Here, we discussed the accuracy of chest PoCUS to delineate its uses in various settings to detect traumatic lung injuries simultaneously which is less discussed in previous studies. Furthermore, the false-negative results are discussed to ascertain the ambiguous situations when decision-making is critical to determine patient disposition in obviously stable or severely unstable patients.

### Analysis of false-negative results and other shortcomings of lung US

#### Pneumothorax

In this study, US sensitivity was lower though closely consistent with other studies suggesting that point-of-care ultrasonography is an acceptable modality to detect pneumothorax. Hyancinthe et al. retrospectively assessed the diagnostic accuracy of US and reported that it can detect PTX and contusion although it was not superior to CXR and chest exam [11]. Furthermore, a review article evaluated US sensitivity in blunt trauma patients and concluded that US is a sensitive screening test to assess PTX [12]. Ianniello et al. estimated the accuracy of US as the first diagnostic modality in major chest trauma patients and found a sensitivity, specificity, PPV and NPV of 77%, 99.8%, 98.5% and 97%, respectively [13]. Lichtenstein et al. showed that the absence of pleural sliding alone has the highest sensitivity (100%) and the presence of lung point provide 100% specificity to detect pneumothorax although Markota has recognized the false-positive absence of pleural sliding due to high positive end expiratory airway pressure [14, 15].

Although the presence of subcutaneous emphysema can be a clue to suspect PTX, it is known to be disruptive to lung ultrasound, scattering US signals [16]. Occult PTX, defined as a very small PTX that can only be found by CT scan, may not be clinically significant [17]. It is not thoroughly evident whether ultrasonography may miss clinically unimportant pneumothorax. However, there are some data to suggest that ultrasound does not miss clinically important PTX [18].

Several studies have discussed the pitfalls of chest sonography to discover pneumothorax. Some researchers described cases with clinically significant pneumothorax who were not diagnosed by chest ultrasonography due to the uneven distribution of pneumothorax in the pleural cavity with pulmonary adhesions to the chest wall with or without lung contusion. Moreover, “double lung point sign” as reported, may under or overestimate the PTX size by ultrasonography [4, 7, 19]. The extent of PTX was estimated by Blaivas et al. assessing the pleural sliding in several intercostal spaces and identified fair correlation with CT scan findings. They also evaluated pleural sliding by standard scan areas of “Power Doppler” to detect the fine pleural shimmering that may be difficult to detect due to resolution limitations [20, 21]. Some suggested differential diagnoses of dyspnea account for the most false-positive pneumothoraces in intensive care units including lung cancer, significant pulmonary infiltration or contusion, pleural or pulmonary adhesions and thoracic surgery, and in other settings such as achalasia [7, 22], many of which are grouped in Table 4.

#### Hemothorax

US has provided high specificity despite lower sensitivity in our study compared with other studies in the assessment of hemothorax that can be due to small insignificant amounts of HTX. In this context, Hyacinthe et al. reported that missed hemothoraces can be due to minimal amounts of blood or those located posteriorly. They mentioned two missed cases suffering from concomitant subcutaneous emphysema and thus reported a modest accuracy for US to assess hemothorax, nearly similar to CXR [11]. Chest X-ray has been known to have low sensitivity in clinically significant injuries [23]. Besides, hemothorax can be falsely recognized in cases of pleural effusion of other causes. However, ultrasonography has shown appropriate sensitivity (92%) and specificity (100%) in the evaluation of hemothorax in trauma patients by Brooks et al. [24]. Also, Atkinson showed that US has sensitivity 92–100% and specificity 100% to detect hemothorax in intensive care settings [25]. Table 4 shows the possible suggested causes of false US results.

#### Pulmonary contusion

The sensitivity of PoCUS was rather low in our study that may be caused by the inclusion of other lung injuries simultaneously. Leblanc D et al. showed that PoCUS can predict acute respiratory distress syndrome by detecting lung contusion in the first 72 h after trauma (receiving operator characteristic curve − area under the curve = 0.78 [95% CI 0.64–0.92]). The extent of contusion diagnosed by US had fair correlation with the corresponding clinical findings [26]. Trying to find various signs of lung contusion can improve the US diagnostic sensitivity. Some researchers reported high accuracy of the US in detecting alveolo-interstitial syndrome (AIS) (95.4%), although the diagnostic accuracy was estimated to be 65.9% in peripheral parenchymal lesions (PPL) [27]. Moreover, Helmy et al. assessed lung contusion by US to have 97.5% sensitivity, 90% specificity, PPV 97.5% and NPV 90% detecting AIS and PPL images [4]. Lung contusion may be falsely diagnosed in patients with acute respiratory distress syndrome (ARDS) and cardiogenic pulmonary edema by ultrasonography. In other words, ultrasonography may not be decisive to definitely distinguish pulmonary contusion and ARDS as contusion may be a predisposing factor and a severity determinant for acute respiratory distress syndrome and further complications [28–31]. The mechanism, severity and the time elapsed from the injury may be clinically helpful [32]. Further studies can address this issue as the current evidence require larger sample sizes [33]. Contusion can be falsely interpreted in the presence of a large PTX due to the presence of a collapsed lung. In addition, the falsely missed lung injuries did not correlate with concomitant systemic injuries in poly-trauma patients if it serves as a distracting factor for the US operator and this issue was consistent with our findings. Also, subcutaneous emphysema and injury in zones not accessible by US may lead to underdiagnose contusion [11]. Soldati et al. reported two patients with documented pulmonary fibrosis among the missed lung contusions in ultrasonography [27].

Overall, considering several criteria for the detection of pneumothorax increase the accuracy of ultrasonography rather than each, including pleural sliding, seashore sign, and normal A and B lines. It seems that the false-negative results of ultrasonography for the diagnosis of traumatic pneumothorax can be compensated by the findings of positive physical examination especially chest wall pain, decreased lung sounds and subcutaneous emphysema. Nonetheless, ultrasonography alone did not show convincing results regarding the false-negative cases of hemothorax and contusion. The possible cause may be the small amounts of blood or pulmonary contusion which can be well-defined in CT scan. Of note, the clinical significance of the detection of small PTX, HTX or contusion is questionable. The beneficial information added by PoCUS to clinical findings serves as a valuable diagnostic guide in emergency situations. In hemodynamically unstable patients not responding to resuscitation, PoCUS can be a useful guide to discover the source of instability. Non-massive hemothorax and contusion can be evaluated and treated after life-threatening conditions are obviated even if missed in the very first critical patient presentation. Additionally, in perfectly stable patients for whom CT scan may be safely deferred, it is beneficial to apply PoCUS as a screening tool to discharge patients safely without the need for CT scan.

## Recommendations and limitations

The accuracy of bedside ultrasonography may vary between intercostal spaces in the diagnosis of pneumothorax. Clinically significant lung injuries were difficult to follow as the subgroup analysis did not acquire sufficient sample size to reach desirable power. Occasionally, the small size of positive samples cause wide confidence intervals that can be further studied with larger sample sizes. This stratification of results based on the ISS, intubated patients, and decreased level of consciousness were not adequately powered and reliable.

The extent of lung contusion can be further studied regarding the hospital observation and long-term outcomes. Furthermore, the lung injuries leading to admission usually coincide with other traumatic injuries that also deserve close monitoring, hospital admissions, surgery or intubation and the differentiation between the exact leading causes was often difficult. In addition, there is a concern about a simple pneumothorax to expand and evolve into tension pneumothorax due to positive pressure ventilation and this issue can be addressed comparing US and CT scan in the future.

## Conclusion

The diagnostic performance of point-of-care ultrasonography was sensitive for blunt pneumothorax and can be beneficial for making disposition of stable patients and helpful in detecting PTX in unstable patients. POCUS was a moderately appropriate modality for diagnosing hemothorax and lung contusion compared to the gold standard, CT scan. Physical exam and ultrasonography augment the diagnostic accuracy of detecting traumatic lung injuries. Pitfalls and caveats should be considered to interpret lung ultrasound in specific situations regarding false-negative and false-positive instances.

## Data Availability

None.

## Abbreviations

PGY-3: Post-graduate year-3
PoCUS: Point-of-care ultrasonography
PPV: Positive-predictive value
NPV: Negative-predictive value
CI: Confidence interval
ATLS: Advanced trauma life support
PTX: Pneumothorax
HTX: Hemothorax
CT: Computed tomography
CXR: Chest X-ray
eFAST: Extended focused assessment with sonography in trauma
AIS: Alveolo-interstitial syndrome
PPL: Peripheral parenchymal lesions
US: Ultrasonography
ARDS: Acute respiratory distress syndrome
